# SARS-CoV-2 and autoantibodies in the cerebrospinal fluid of COVID-19 patients: prospective multicentre cohort study

**DOI:** 10.1093/braincomms/fcad274

**Published:** 2023-10-17

**Authors:** Vardan Nersesjan, Moshgan Amiri, Anna Christine Nilsson, Christian Wamberg, Veronika Vorobieva Solholm Jensen, Charlotte Bjerg Petersen, Anne-Mette Hejl, Anne-Mette Lebech, Anna Marie Theut, Charlotte Sværke Jørgensen, Morten Blaabjerg, Michael E Benros, Daniel Kondziella

**Affiliations:** Biological and Precision Psychiatry, Copenhagen Research Center for Mental Health, Mental Health Centre Copenhagen, Copenhagen University Hospital, Hellerup 2900, Denmark; Department of Neurology, Rigshospitalet, Copenhagen University Hospital, Copenhagen 2100, Denmark; Faculty of Health and Medical Sciences, Department of Immunology and Microbiology, University of Copenhagen, Copenhagen 2200, Denmark; Department of Neurology, Rigshospitalet, Copenhagen University Hospital, Copenhagen 2100, Denmark; Department of Clinical Immunology, Odense University Hospital, Odense 5000, Denmark; Department of Anesthesia and Intensive Care, Bispebjerg and Frederiksberg Hospital, Copenhagen University Hospital, Copenhagen 2400, Denmark; Virus and Microbiological Special Diagnostics, Statens Serum Institut, Copenhagen S 2300, Denmark; Department of Neurology, Bispebjerg and Frederiksberg Hospital, Copenhagen University Hospital, Copenhagen 2400, Denmark; Department of Neurology, Bispebjerg and Frederiksberg Hospital, Copenhagen University Hospital, Copenhagen 2400, Denmark; Department of Infectious Diseases, Rigshospitalet, Copenhagen University Hospital, Copenhagen 2100, Denmark; Department of Clinical Medicine, University of Copenhagen, Copenhagen 2200, Denmark; Virus and Microbiological Special Diagnostics, Statens Serum Institut, Copenhagen S 2300, Denmark; Virus and Microbiological Special Diagnostics, Statens Serum Institut, Copenhagen S 2300, Denmark; Department of Neurobiology Research, Institute of Molecular Medicine, University of Southern Denmark, Odense 5000, Denmark; Biological and Precision Psychiatry, Copenhagen Research Center for Mental Health, Mental Health Centre Copenhagen, Copenhagen University Hospital, Hellerup 2900, Denmark; Department of Neurology, Rigshospitalet, Copenhagen University Hospital, Copenhagen 2100, Denmark; Department of Neurology, Rigshospitalet, Copenhagen University Hospital, Copenhagen 2100, Denmark; Department of Clinical Medicine, University of Copenhagen, Copenhagen 2200, Denmark

**Keywords:** COVID-19, CSF, SARS-CoV-2, encephalitis, autoantibodies

## Abstract

Disease mechanisms underlying neurological and neuropsychiatric symptoms after coronavirus disease 2019 (COVID-19), termed neuro-COVID, are poorly understood. Investigations of the cerebrospinal fluid (CSF) for the presence of severe acute respiratory syndrome coronavirus 2 (SARS-CoV-2) RNA and antibodies, as well as autoantibodies against neuronal surface antigens, could improve our understanding in that regard. We prospectively collected CSF and blood from patients investigated by lumbar puncture for neurological or neuropsychiatric symptoms during or after COVID-19. Primary outcomes were the presence of (i) SARS-CoV-2 RNA in CSF via polymerase chain reaction (PCR), (ii) SARS-CoV-2 immunoglobulin G (IgG) anti-S receptor-binding-domain antibodies via the Euroimmun and Wantai assays and (iii) IgG autoantibodies against neuronal surface antigens using commercial cell- and tissue-based assays (Euroimmun). Secondary outcomes were (i) routine CSF investigations and (ii) correlation between SARS-CoV-2 antibody levels in CSF with serum levels, blood–brain barrier permeability and peripheral inflammation. We obtained CSF from 38 COVID-19 patients (mean age 56.5 ± 19.2 years, 53% women) who developed neurological and neuropsychiatric symptoms. CSF pleocytosis (>5 cells) was observed in 9/38 patients (23.7%), elevated CSF protein (>0.50 g/L) in 13/38 (34.2%) and elevated CSF/serum albumin ratio in 12/35 (34.3%). PCR for SARS-CoV-2 RNA in CSF was negative in all. SARS-CoV-2 CSF antibodies were detected in 15/34 (44.1%; Euroimmun assay) and 7/31 (22.6%; Wantai assay) individuals, but there were no signs of intrathecal SARS-CoV-2 IgG production. SARS-CoV-2 CSF antibodies were positively correlated with serum levels (*R* = 0.93, *P* < 0.001), blood–brain barrier permeability (*R* = 0.47, *P* = 0.006), peripheral inflammation (*R* = 0.51, *P* = 0.002) and admission to the intensive care unit [odds ratio (OR) 17.65; 95% confidence interval (CI) 1.18–264.96; *P* = 0.04; *n* = 15]. Cell-based assays detected weakly positive NMDAR, LGI1 and CASPR2 antibodies in serum of 4/34 (11.8%) patients but not in CSF. The tissue-based assay showed anti-neuronal fluorescence in CSF from one individual, staining for Purkinje cells. In summary, whereas we did not detect active SARS-CoV-2 infection in the CSF, SARS-CoV-2 antibodies were prevalent. The absence of intrathecal antibody production points towards blood–brain barrier impairment as the origin of CSF SARS-CoV-2 antibodies. In contrast, CSF autoantibodies against neuronal surface antigens were rare. There was no evidence for a clinical correlate of these antibodies. We conclude that, rather than specific autoimmune neuronal injury, non-specific effects of critical illness including an impaired blood–brain barrier are more likely to contribute to neuro-COVID.

## Teaser

CSF was analysed from 38 neuro-COVID patients. None had SARS-CoV-2 RNA in CSF. Forty-four per cent exhibited CSF anti-spike SARS-CoV-2 antibodies without signs of intrathecal synthesis, but antibody levels correlated strongly with blood–brain barrier permeability. CSF anti-neuronal autoantibodies were not detected. Therefore, critical illness and peripheral inflammation contribute mostly to neuro-COVID.

## Graphical Abstract

**Graphical Abstract:**
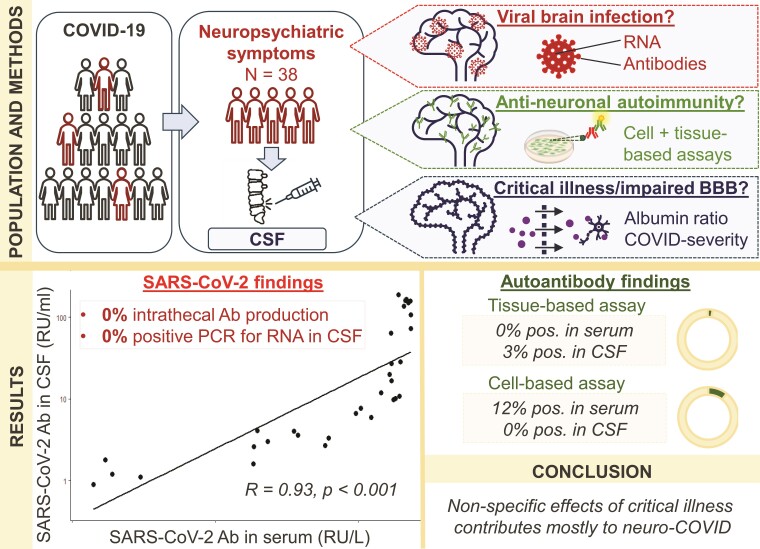

## Introduction

Neurological and neuropsychiatric symptoms following coronavirus disease 2019 (COVID-19) are common,^1-4^ but the underlying pathophysiology is poorly understood. In the early phase of the pandemic, a post-mortem study^5^ and case reports^6,7^ revealed severe acute respiratory syndrome coronavirus 2 (SARS-CoV-2) RNA in neuronal tissue, sparking interest in possible neurotropism of SARS-CoV-2 as a potential cause of the neurological and neuropsychiatric manifestations of neuro-COVID.^8^ However, evidence of SARS-CoV-2 neurotropism could not be replicated in subsequent post-mortem studies,^9,10^ nor was SARS-CoV-2 RNA detected in the cerebrospinal fluid (CSF) of most patients investigated.^11-13^ Although SARS-CoV-2 CSF antibodies appear to occur more frequently, and specific anti-SARS-CoV-2 immunoglobulin G (IgG) intrathecal synthesis can indeed occur,^14^ the observed SARS-CoV-2 antibodies in the CSF are likely an epiphenomenon related to an increased permeability of the blood–brain barrier (BBB).^15-18^

Infections can induce autoimmune reactivity, and anti-neuronal autoantibodies in COVID-19 have been reported in case reports,^19-21^ but COVID-19-associated autoimmune encephalitis is even rarer.^22^ A small CSF study from critically ill COVID-19 patients revealed neuronal cross-reactivity to unknown antigen epitopes,^23^ yet the clinical implications are unknown. In contrast, the largest CSF COVID-19 study to date (*n* = 127)^24^ did not reveal any positive results with indirect immunofluorescence using cerebellum brain sections, but data were only reported for a subgroup of patients (*n* = 34) and the clinical generalizability is limited owing to the study’s retrospective nature. Prospective cohorts of both mildly affected and critically ill COVID-19 patients investigated for anti-neuronal autoantibodies and SARS-CoV-2 antibodies in the CSF are required to elucidate the mechanisms behind COVID-related CNS autoimmunity and, potentially, viral neuroinvasion.

Here, we investigated SARS-CoV-2 RNA and antibodies, as well as anti-neuronal autoantibodies, in the CSF of COVID-19 patients with neurological or neuropsychiatric symptoms in a prospective multicentre cohort study. Specifically, we aimed to investigate whether SARS-CoV-2 antibodies are produced intrathecally or are indicative of BBB leakage and whether known anti-neuronal antibodies can be detected via cell- and brain-tissue-based assays.

## Materials and methods

### Study design and population

This multicentre prospective study was a collaboration between the neurological, infectious diseases and intensive care departments at three academic hospitals in Copenhagen in Denmark (Rigshospitalet, a tertiary referral centre; CSF samples from *n* = 17 patients; Bispebjerg Hospital, *n* = 19; and Herlev Hospital, *n* = 2), from April 2020 to December 2021. The study was approved by the Regional Ethics committee (H-20026602) and Data Protection Agency (P-2020-497) of the Capital Region of Denmark. Verbal and written consents were obtained from all participants or legal next of kin. Patients were enrolled when they were investigated as part of routine clinical work-up with a lumbar puncture for neurological or neuropsychiatric symptoms that developed during or after COVID-19. Inclusion criteria were: (i) age ≥18 years; (ii) positive SARS-CoV-2 polymerase chain reaction (PCR) test in pharyngeal or tracheal testing and (iii) new-onset neurological or neuropsychiatric symptoms attributed by the attending clinicians to a previous or an active SARS-CoV-2 infection. Blood and CSF were investigated for the presence of SARS-CoV-2 RNA and SARS-CoV-2 antibodies against the receptor-binding-domain of the spike protein and for the presence of anti-neuronal autoantibodies. Patient data were collected during clinical work-up, including neurological investigations. We collected clinical and laboratory data, including sex, age, body mass index, comorbidities and medical history, data regarding hospitalization including intensive care unit (ICU) admission, laboratory results and neuroimaging findings. Study participants were followed for up to 1 year after the onset of neurological and/or neuropsychiatric symptoms through electronic healthcare records, and the trajectory of symptoms was categorized as ‘spontaneous remission’, ‘remission after treatment’, ‘persistent symptoms at last follow-up’ and ‘died with symptoms’. Participants were grouped according to WHO criteria in *non-severe*, *severe* or *critical* COVID-19.^25^ Patients were categorized in three main clinical phenotypes based on prior used definitions,^1,26^ i.e. central nervous system (CNS) affection, peripheral nervous system (PNS) affection and acute or prolonged neuropsychiatric symptoms. CNS affection included *cerebrovascular* (ischaemic and haemorrhagic confirmed via neuroimaging); *encephalopathy* (including disorders of consciousness after sedation stop in the ICU) based on consensus criteria for encephalopathy^27^ and *neuroinflammation* (including encephalitis,^28^ CNS vasculitis^29^ and myelitis^30^). PNS affection encompassed peripheral facial palsy and critical illness polyneuromyopathy (CIMP). Acute neuropsychiatric symptoms (onset within 2 weeks after a positive SARS-CoV-2 PCR test) included headache, fatigue and confusion that led to a neurological investigation and lumbar puncture. Prolonged neuropsychiatric symptoms included fatigue, headache, confusion, aphasia, vertigo, memory problems, concentration difficulties, anxiety and/or depression lasting >3 months^31^ after COVID-19 onset and led to a neurological investigation and lumbar puncture. One patient with encephalopathy was diagnosed with cerebral autosomal dominant arteriopathy with subcortical infarcts and leucoencephalopathy (CADASIL). Clinical data of three patients (CADASIL, encephalitis and myelitis) have been published previously.^1^

### Outcomes

Primary outcomes were (i) quantified levels of SARS-CoV-2 antibodies in CSF, (ii) presence of SARS-CoV-2 RNA in CSF and (iii) detection of any anti-neuronal antibodies against cell-surface antigens using commercial assays.

Secondary outcomes were (i) CSF inflammation, i.e. pleocytosis (>5 cells) and protein levels (>0.50 g/L), (ii) the correlation between SARS-CoV-2 antibodies in the CSF with the albumin ratio and serum leucocytes and (iii) the association of severe COVID-19 with the presence of SARS-CoV-2 antibodies in the CSF.

### Outcome measurements

#### Routine findings and blood–brain barrier dysfunction

Total CSF leucocyte counts were dichotomized in pleocytosis (>5 cells) and normal. Upper threshold for normal CSF protein levels was set at 0.50 g/L.^32^ The BBB was assessed using the CSF (mg/L)/serum (g/L) albumin quotient calculated as Q-Alb = Alb-CSF_(mg/L)_/Alb-serum_(g/L)_. Upper limit threshold (Q-limAlb) for Q-Alb in each individual patient was calculated as (4 + (*a*/15)) with *a* representing the patient’s age according to Reiber *et al*.^33,34^ A dysfunctional BBB was defined as Q-Alb > Q-limAlb.^33,34^

#### SARS-CoV-2 PCR and antibody evaluation

The qualitative Wantai SARS-CoV-2 Total Antibody ELISA (Beijing Wantai Biological Pharmacy Enterprise),^35^ and the quantitative assay, the anti-SARS-CoV-2 QuantiVac ELISA (IgG; Euroimmun Medizinische Labordiagnostika AG)^36^ kits were used for measuring anti-SARS-CoV-2 antibodies in CSF and paired serum samples according to the manufacturers’ instructions. The Wantai assay is based on a double-antigen sandwich principle that detects total antibodies binding to the SARS-CoV-2 spike protein receptor-binding domain. IgG antibodies against SARS-CoV-2 spike protein Subunit 1, including the receptor-binding domain, are detected by the assay from EUROIMMUN. Serum and CSF samples were PCR screened for SARS-CoV-2 RNA; herpes simplex virus (HSV-1 and 2) and varicella-zoster virus DNA and enterovirus RNA.

#### SARS-CoV-2 immunoglobulin G intrathecal synthesis

To determine SARS-CoV-2-specific intrathecal synthesis, an antibody index (AI) was calculated using Reiber’s formula^37^ as previously described.^16,24^

The specific SARS-CoV-2 quotient (Q-SARS) was Q-SARS = IgG(SARS)-CSF/IgG(SARS)-serum, and the total IgG quotient (Q-IgG) was Q-IgG = IgG-CSF/IgG-serum. The intrathecal index was calculated as AI = Q-SARS/Q-IgG. If Q-IgG exceeded Q-limIgG, a proposed^33^ correction was applied and AI was calculated as AI = Q-SARS/Q-limIgG. Q-limIgG was calculated as: Q-limIgG = (0.93 [Q-Alb^2^ + 6 × 10^6^]^0.5^ − 1.7) × 10^−3^.^16^ AI values >1.5 were considered positive and a sign of specific SARS-CoV-2 intrathecal synthesis.^33^

#### Autoantibody assessment using commercial cell- and tissue-based assays

Anti-neuronal antibodies (NMDAR NR1 subunit, CASPR2, LGI1, AMPAR1, AMPAR2, GAD65 and GABAb receptor B1/B2) were investigated by indirect immunofluorescence using commercial cell-based assay (Euroimmun, Lübeck, Germany), according to the manufacturer’s recommendations. Serum samples were analysed in dilution 1:10, and CSF samples were analysed undiluted. Results were reported according to fluorescence intensity as negative (lack of specific fluorescence), borderline positive, weakly positive, moderately positive and strongly positive. Additionally, serum (dilution 1:10 and 1:100) and CSF (undiluted) samples were analysed via commercial tissue-based assay (Euroimmun) using monkey cerebellum sections according to the manufacturer’s recommendations. Samples were reported positive if any anti-neuronal antibodies were detected, and fluorescence intensity was reported. Samples positive by tissue-based assay were also tested using a commercial line blot; EUROLINE PNS 12 Ag (Euroimmun).

### Statistical analysis

Outcomes were analysed with complete-case analysis without imputation (supplementary Table S1). Continuous variables of baseline characteristics and primary and secondary outcomes were assessed via histograms and Q-Q plots to determine distribution. All data except for body mass index were non-normally distributed; therefore, non-parametric statistical methods were used in analysis of continuous data. Patient population was dichotomized based on the severity of the initial COVID-19 infection in non-severe cases and in severe or critical cases based on the WHO established criteria.^25^ Baseline characteristics were compared between the dichotomized groups using Pearson’s χ^2^ test (categorical variables) and Wilcoxon rank-sum test (continuous variables). Primary outcomes of SARS-CoV-2 investigations were calculated as prevalence of positive or negative findings of SARS-CoV-2 RNA and antibodies (using manufacturer’s cut-off for antibody data) in serum and CSF, and quantified levels of SARS-CoV-2 antibodies were calculated as medians with interquartile range (IQR) and compared across groups of COVID-19 severity (*non-severe*, *severe* and *critical*) using Kruskal–Wallis one-way analysis of variance and Bonferroni corrected (three groups) Wilcoxon rank-sum test. Primary outcome of anti-neuronal antibody findings using commercial cell- and tissue-based assays were calculated as total prevalence. For secondary outcomes, quantified levels of SARS-CoV-2 antibodies in CSF were correlated with corresponding levels in serum, with Q-Alb and peripheral leucocyte levels using Spearman’s rank correlation coefficient. The presence of SARS-CoV-2 antibodies in CSF was further analysed in a logistic regression model with covariates of age, sex, COVID-19 severity and Q-Alb. A two-tailed *P*-value <0.05 was considered significant. Data were analysed using R, version 4.1.2.

## Results

### Demographics and clinical characteristics

Thirty-eight SARS-CoV-2 PCR-positive COVID-19 patients [mean age (SD) 56.5 (19.5) years, 53% women] were included for investigations and either developed a CNS or PNS complication (*n* = 28) or suffered from acute or prolonged neuropsychiatric symptoms (*n* = 9) during or after COVID-19, while one individual had undetermined acral paresthesia with onset before COVID-19 (see Table 1 for demographics and clinical characteristics).

**Table 1:** Demographics and clinical characteristics of COVID-19 patients with neurological and neuropsychiatric symptoms

		COVID-19 severity		
	Total *N* = 38	Non-severe *N* = 12	Severe or critical *N* = 26	*P*-value
Age, mean (SD), years	56.5 (19.5)	37.8 (13.8)	65.1 (15.2)	<0.001
Sex, female, *n* (%)	20 (52.6)	7 (58.3)	13 (50.0)	0.898
BMI, mean (SD)	24.4 (4.91)	22.9 (2.42)	25.0 (5.50)	0.304
Medical history, *n* (%)				
Hypertension	13 (34.2)	0 (0)	13 (50)	0.008
Hyperlipidaemia	6 (15.8)	1 (8.3)	5 (19.2)	0.706
Malignancy	3 (7.9)	1 (8.3)	2 (7.7)	>0.99
Asthma	3 (7.9)	1 (8.3)	2 (7.7)	>0.99
COPD	4 (10.5)	0 (0)	4 (15.4)	0.385
Stroke	3 (7.9)	0 (0)	3 (11.5)	0.563
Depression	2 (5.3)	0 (0)	2 (7.7)	0.837
Anxiety	1 (2.6)	1 (8.3)	0 (0)	0.688
COVID-19 clinical characteristics, *n* (%)				
WHO disease severity				
Non-severe	12 (31.6)			NA
Severe	11 (28.9)			NA
Critical	15 (39.5)			NA
Hospitalization for COVID-19	27 (71.1)	0 (0)	26 (100)	<0.001
ICU admission	15 (39.5%)		15 (57.7)	NA
Days in ICU, median (IQR)	30.0 (19.0–47.5)		30.0	NA
Mortality during hospitalization	6 (15.8)		6 (23.1)	NA
Neurological phenotype at time of lumbar puncture, *n* (%)				
CNS affection	20 (52.6%)	2	18	0.003^a^
Cerebrovascular	5	0	5	
Neuroinflammation	4	2	2	
Encephalopathy	11	0	11	
PNS affection	8 (21.1%)	2	6	
Peripheral facial palsy	5	2	3	
CIMP	3	0	3	
Acute neuropsychiatric symptoms^b^	3 (7.9%)	2	1	
Prolonged neuropsychiatric symptoms^b^	6 (15.8%)	5	1	
Days from positive SARS-CoV-2 PCR to neurological symptom debut, median (IQR)	16.0 (6.0–27.0)	13.0	19.5	0.405
Days from positive SARS-CoV-2 PCR to CSF collection, median (IQR)	26.5 (16.5–48.5)	24.0	27.0	0.962

BMI, body mass index; COPD, chronic obstructive pulmonary disease; CNS, central nervous system; CIMP, critical illness myo-polyneuropathy; CSF, cerebrospinal fluid; ICU, intensive care unit; IQR, interquartile range; NA, not applicable; PNS, peripheral nervous system; PCR, polymerase chain reaction; SD, standard deviation.

^a^ *P*-value was calculated for the total association of CNS and PNS symptoms and acute or prolonged neuropsychiatric symptoms between mild and severe/critical COVID-19 using the Kruskal–Wallis one-way analysis of variance.

^b^At the time of lumbar puncture: Acute (debut < 2 weeks after COVID-19 onset) and prolonged (persisting > 3 months after COVID-19) neuropsychiatric symptoms including headache, fatigue, confusion, aphasia, vertigo, memory problems, concentration difficulties, anxiety and/or depression, leading to a neurological investigation and lumbar puncture.

Twenty-seven of 38 (71.1%) patients were hospitalized for COVID-19, of whom 15 (56%) were admitted to the ICU. The ICU cohort consisted mainly of encephalopathic patients investigated for a CNS infection and of patients with CIMP investigated for Guillain–Barre syndrome, but we did not find any indications for autoimmunity underlying their symptoms. COVID-19 was non-severe in 12 of 38 (31.6%), severe in 11 of 38 (28.9%) and critical in 15 of 38 (39.5%) patients. Median time from positive SARS-CoV-2 PCR to the onset of neurological symptoms was of 26.5 (IQR: 16.5–48.5) days, and median time from neurological symptom debut to lumbar puncture was 6 (IQR: 2–20) days.

### Clinical trajectories 1 year after onset of neurological/neuropsychiatric symptoms

Ten of 38 (18.4%) had a spontaneous remission. Two of 38 (5.3%) had a remission after immunosuppressive treatment for a transverse myelitis and primary CNS vasculitis, respectively, although the patient with CNS vasculitis had one relapsing episode. Fourteen of 38 (36.8%) patients had persisting neuropsychiatric symptoms 1 year after COVID-19, including 5 (35.7%) with persisting cognitive and neuropsychiatric symptoms after encephalopathy during admission, 3 (21.4%) with severe neurological deficits after stroke, 2 (14.3%) with persistent focal neurological deficits after encephalitis and myelitis despite immunosuppressive treatment, 2 (14.3%) with persistent neuropathic deficits after critical illness polyneuropathy/myopathy and 2 (14.3%) with persistent neuropsychiatric symptoms and severe fatigue after mild COVID-19. In total, 8 of 38 (21.1%) patients died with neurological symptoms, including 4 comatose patients who died without having regained consciousness. The remaining 3 of 38 had no further contact with the healthcare system besides the initial investigation.

### Routine cerebrospinal fluid findings and blood–brain barrier dysfunction

CSF leucocytes were elevated (>5 cells) in 9 of 38 (23.7%), and CSF protein was elevated (>0.50 g/L) in 13 of 38 (34.2%) patients. Using Reiber’s age dependent cut-off, BBB dysfunction was evident in 12 of 35 (34.3%) patients.

### SARS-CoV-2 investigations

#### PCR for viral RNA

PCR for SARS-CoV-2 RNA was positive in 1 of 30 (3.3%) serum samples but in none of the CSF samples (0/30). PCR for HSV-1 was strongly positive in 2 of 30 (6.6%) serum samples and weakly positive in 1 of 30 (3.3%) CSF samples. The latter CSF sample was only positive in 1 of 4 tested samples, but subsequent investigations were negative for intrathecal HSV-1 antibody production, and the patient had no symptoms of a herpes encephalitis; thus, this result was deemed false-positive. PCR for HSV-2 was negative in all serum samples but weakly positive in 1 of 30 (3.3%) CSF samples from an encephalopathic patient who died shortly after investigation. All serum or CSF samples were PCR negative for enterovirus.

#### SARS-CoV-2 antibody findings

**Figure 1:**
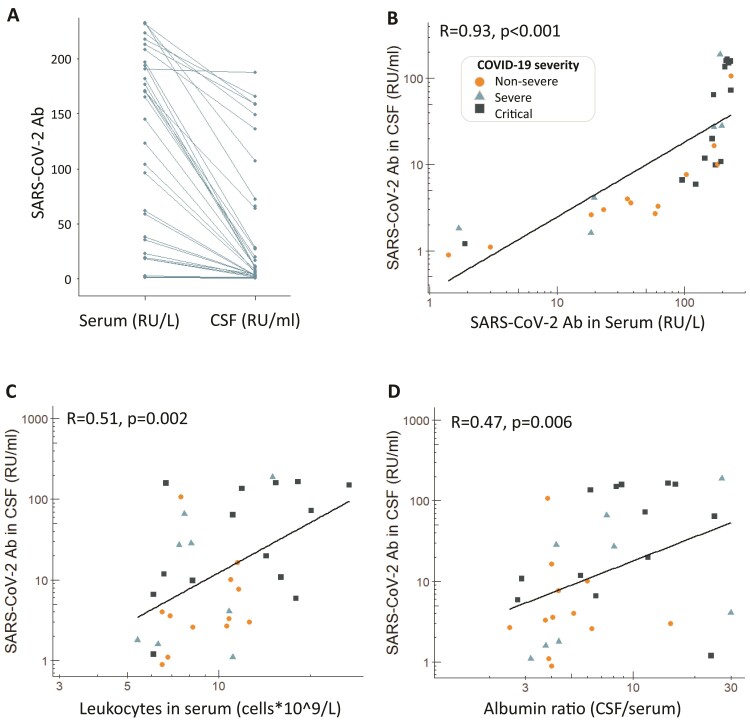
**SARS-CoV-2 antibodies in relation to COVID-19 severity, albumin ratio and peripheral inflammation.** Total levels of SARS-CoV-2 antibodies against the spike protein S1 in serum and CSF are depicted (**A**). Using Spearman’s rank correlation, a significant positive correlation is seen between (**B**) serum and CSF SARS-CoV-2 antibodies against the spike protein S1; and between CSF SARS-CoV-2 antibodies and (**C**) leucocytes in serum and (**D**) the albumin ratio (CSF/serum).

**Figure 2:**
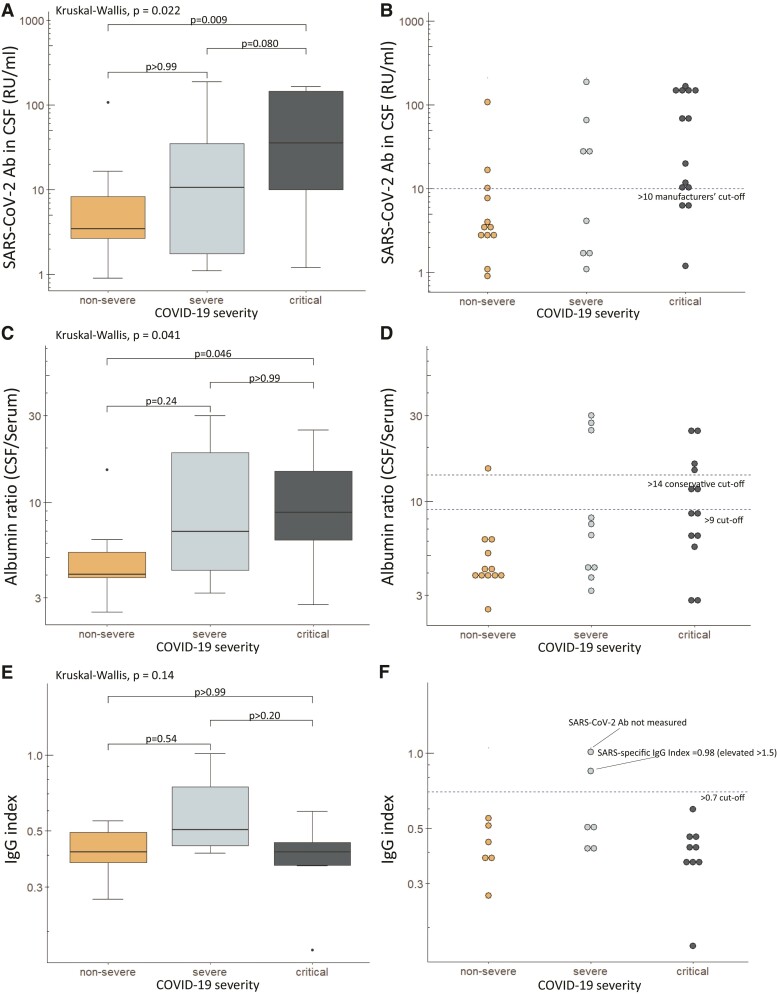
**Levels of SARS-CoV-2 CSF antibodies, albumin ratio and IgG index in patients with non-severe, severe and critical COVID-19.** Box plots and dot plots are depicted in patients with non-severe, severe and critical COVID-19, showing levels of CSF SARS-CoV-2 antibodies against the spike protein S1 (**A, B**); the albumin ratio (CSF/serum) (**C, D**) and the IgG index (**E-D**). The dashed line in (**B**) shows the manufacturers’ recommended upper threshold for non-infected individuals (i.e. negative finding^36^); in (**D**), the cut-off >9 is based on Danish guidelines^38^ and >14 as a more conservative choice; and in (**F**), the cut-off >0.7 indicates possible intrathecal IgG synthesis.^39^ One patient with IgG index >0.7 had no SARS-CoV-2 antibody testing (although PCR negative in CSF) and the other patient had a SARS-specific IgG index <1.5 which was considered the threshold for specific SARS-CoV-2 intrathecal synthesis.^33^ Kruskal–Wallis one-way analysis of variance was used to compare >2 subgroups, and a Wilcoxon rank-sum test with Bonferroni correction (three groups) was applied to further investigate which subgroups differed from each other. The *P*-values shown in the figure are adjusted values after correcting for multiple testing.

The Wantai total SARS-CoV-2 antibody assay was positive in 30 of 31 (96.7%) serum samples and in 7 of 31 (22.6%) CSF samples. The Euroimmun SARS-CoV-2 IgG antibody assay yielded 28 positive results out of 32 (87.5%) serum samples and 15 out of 34 (44.1%) CSF samples, but no patient had SARS-CoV-2 specific intrathecal IgG synthesis. Quantified levels (RU/mL) of SARS-CoV-2 antibodies via the Euroimmun assay were higher in the serum versus CSF [median (IQR): 155.20 (32.50–194.78) versus 9.95 (3.08–65.53), *P* < 0.001], and there was a strong linear relationship between serum and CSF antibody levels (*R* = 0.93, *P* < 0.001), with the QAlb reflecting BBB permeability (*R* = 0.47, *P* = 0.006) and with leucocyte levels in blood reflecting peripheral inflammation (*R* = 0.51, *P* = 0.002; Fig. 1). Overall, median (IQR) SARS-CoV-2 antibodies in the CSF was highest in patients with critical COVID-19; 41.98 (10.05–146.05), followed by severe COVID-19; 15.55 (1.75–37.65) and non-severe COVID-19; 3.45 (2.68–8.30), *P* = 0.02; and specifically, the significant difference was between patients with non-severe versus critical COVID-19 (Bonferroni corrected *P* = 0.009) while differences between non-severe versus severe and severe versus critical were non-significant. Two patients had an elevated IgG index (>0.7), indicating intrathecal IgG synthesis, but one had no SARS-CoV-2 antibodies measured (encephalitis with negative SARS-CoV-2 PCR in CSF) and the other had a SARS-specific intrathecal index of 0.98 (cut-off >1.5; see Fig. 2). In our regression model, critical COVID-19 requiring ICU admission was associated with increased odds of SARS-CoV-2 antibodies in the CSF (OR 17.65, 95% CI 1.18–264.96, *P* = 0.04; Table 2).

**Table 2:** Routine and microbiological investigations of serum and cerebrospinal fluid from COVID-19 patients with neurological and neuropsychiatric symptoms

		COVID-19 severity		
	Total *N* = 38	Non-severe *N* = 12	Severe or critical *N* = 26	*P*-value
Routine CSF analysis				
Leucocytes, (cells 10^6^/L), median (IQR)	2.9 (1.3–3.8)	2.9 (2.0–3.0)	2.5 (1.0–6.3)	0.489
Pleocytosis (>5 cells), *n*/*N* (%)	9/38 (23.7)	2/12 (16.7)	7/26 (26.9)	0.779
Total leucocytes from patients with >5 cells, median (range)	10 (7–271)	67.5 (10–125)	8 (7–271)	0.460
Total protein, g/L, median (IQR)	0.41 (0.29–0.56)	0.31 (0.28–0.37)	0.47 (0.34–0.58)	0.076
Elevated protein (>0.50 g/L),^32^*n*/*N* (%)	13/38 (34.2)	2/12 (16.7)	11/26 (43.5)	0.123
Albumin, mg/L, median (IQR)	201 (148–292)	158 (144–202)	227 (160–314)	0.132
Albumin CSF_(dg/L)_/serum ratio_(g/L)_, median (IQR)	0.062 (0.04–0.117)	(0.04 (0.039–0.054)	0.081 (0.05–0.155)	0.013
Dysfunctional BBB (QAlb > Qlim), *n*/*N* (%)	12/35 (34.3)	2/12 (16.7)	10/23 (38.5)	0.113
Glucose, CSF/serum ratio, median (IQR)	0.6 (0.5–0.7)	0.6 (0.5–0.6)	0.6 (0.5–0.7)	0.900
SARS-CoV-2 investigations				
SARS-CoV-2 RNA via PCR				
Serum, *n*/*N* (%)	1/30 (3.3)	0/11 (0)	1/18 (5.5)	NA
CSF, *n*/*N* (%)	0/30 (0)	0/12 (0)	0/18 (0)	NA
SARS-CoV-2 IgG against SP (Euroimmun)				
Serum (RU/L), median (IQR)	155.2 (32.5–194.8)	48.4 (22.1–120.6)	173.7 (116.5–209.8)	0.048
CSF (RU/mL), median (IQR)	10.0 (3.1–65.5)	3.5 (2.7–8.3)	23.4 (6.1–120.4)	0.017
CSF Ab positive (cut-off >10 RU/mL), *n*/*N* (%)	15/34 (44.1)	2/12 (16.7)	13/22 (59.1)	0.017
SARS-CoV-2 Ab index (CSF/serum), median (IQR)	0.14 (0.08–0.63)	0.11 (0.07–0.20)	0.26 (0.09–0.67)	0.083
SARS-CoV-2 Intrathecal synthesis, *n*/*N* (%)	0/34 (0)	0/12 (0)	0/22 (0)	NA
Viral screening via PCR in CSF				
HSV-1, *n*/*N* (%)	1/30 (3.3)^a^	1/12 (8.3)	0/18 (0)	NA
HSV-2, *n*/*N* (%)	1/30 (3.3)^b^	0/12 (0)	1/18 (5.5)	NA
VZV, *n*/*N* (%)	0/30 (0)	0/12 (0)	0/18 (0)	NA
Enterovirus, *n*/*N* (%)	0/30 (0)	0/12 (0)	0/18 (0)	NA

Ab, antibody; BBB, blood–brain barrier; CSF, cerebrospinal fluid; HSV, herpes simplex virus; IQR, interquartile range; PCR, polymerase chain reaction; QAlb, albumin index (CSF/serum); Qlim, individual age adjusted upper limit for albumin index; SP, spike protein; VZV, varicella-zoster virus.

^a^CSF sample from one patient was HSV-1 positive in one of four tested samples (weakly positive) and subsequently negative for HSV-1 intrathecal antibodies thus deemed not a true positive finding.

^b^CSF sample from one patient was HSV-2 positive in one of four tested samples (weakly positive). No subsequent antibody testing was performed.

### Anti-neuronal antibody investigations

#### Commercial cell- and tissue-based assays

Commercial cell-based assays of serum samples were weakly positive in 4 of 34 (11.8%) patients. Specific antibodies included NMDAR, *n* = 1; LG1, *n* = 2 and CASPR2, *n* = 2 (one individual had both LGI1 and CASPR2 serum antibodies). None of the patients had confirmatory reactions on TBA nor fulfilled consensus criteria for antibody-mediated encephalitis^40^ (supplementary Table 2).

Commercial cell-based assays of CSF samples were negative in all (0 of 38). For the commercial tissue-based assay, one individual [1 of 38 (2.6%)] was positive in CSF showing dotted cytoplasmic staining of Purkinje cells (this sample was tested for anti-Tr/-DNER IgG and anti-Yo IgG, the most common Purkinje cell antibody, but found negative); however, the patient had no cerebellar symptoms (Table 3).

**Table 3:** Anti-neuronal autoantibody investigations via commercial cell- and tissue-based assays

			COVID-19 severity				
	Total *N* = 38		Non-severe *N* = 12		Severe or critical *N* = 26		*P*-value
	CSF	Serum	CSF	Serum	CSF	Serum	
Commercial cell- and tissue-based assay, no. of positive/total tested (%)							
NMDAR	0/38 (0)	1/34 (2.9)^a^	0/12 (0)	0/11 (0)	0/26 (0)	1/23 (4.3)	0.422^b^
CASPR2	0/38 (0)	2/34 (5.9)^a^	0/12 (0)	1/11 (0)	0/26 (0)	1/23 (4.3)	
LGI1	0/38 (0)	1/34 (2.9)^a^	0/12 (0)	1/11 (0)	0/26 (0)	0/23 (0)	
GABA_B_R	0/38 (0)	0/34 (0)	0/12 (0)	0/11 (0)	0/26 (0)	0/23 (0)	
AMPAR-1	0/38 (0)	0/34 (0)	0/12 (0)	0/11 (0)	0/26 (0)	0/23 (0)	
AMPAR-2	0/38 (0)	0/34 (0)	0/12 (0)	0/11 (0)	0/26 (0)	0/23 (0)	
Commercial TBA	1/38 (2.6)^c^	0/34 (0)	0/12 (0)	0/11 (0)	1/26 (3.8)	0/23 (0)	0.491^d^

Findings from commercial cell- and tissue-based assays (Euroimmun) on serum and cerebrospinal fluid samples from COVID-19 patients with neurological and neuropsychiatric symptoms.

AMPAR, alpha-amino-3-hydroxy-5-methyl-4-isoxazolepropionic acid receptor; CSF, cerebrospinal fluid; CASPR2, contactin-associated protein-like 2; GABA-B, gamma aminobutyric acid-B; LGI1, leucin-rich glioma inactivated 1; NA, not applicable; NMDAR, *N*-methyl-d-aspartate receptor; TBA, tissue-based assay.

^a^Serum samples were weakly positive.

^b^ *P*-value is calculated for the comparison of serum findings.

^c^ *P*-value is calculated for the comparison of CSF findings.

^d^CSF sample showing dotted cytoplasmic staining of Purkinje cells. This sample was tested for anti-Tr/-DNER IgG and anti-Yo IgG, which is the most common Purkinje cell antibody, but found negative.

## Discussion

In this prospective study of patients with neurological and neuropsychiatric symptoms associated with mild, severe, or critical COVID-19, SARS-CoV-2 RNA was absent in the CSF, and there were no signs of intrathecal SARS-CoV-2 IgG production. (One patient with encephalitis had elevated IgG index, but SARS-CoV-2 antibody measurements were not available.) We detected antibodies against the SARS-CoV-2 spike protein in the CSF of 22.6–44.1% of the patients, which positively correlated with serum antibody levels, peripheral leucocyte counts, increasing Q-Alb and COVID-19 severity. Utilizing commercial anti-neuronal antibody assays with transfected HEK293 cells, we did not detect known anti-neuronal CSF antibodies.

Anti-neuronal autoantibodies such as NMDAR, LGI-1 and CASPR2 are infrequent in neuro-COVID,^41^ and mainly reported in isolated cases.^19-21^ Few studies have prospectively investigated ‘novel’ autoantibodies via indirect immunofluorescence on whole-brain sections.^23,41,42^ In critically ill COVID-19 patients (*n* = 11), Franke *et al*.^23^ reported strong IgG binding on several antigen epitopes of neuronal, astrocytic and vascular proteins, while a case series of three adolescents with COVID-19 and acute neuropsychiatric symptoms^42^ revealed IgG-CSF staining of mitral cells from the olfactory bulb, cortical neurons and Purkinje cells. In contrast, in a study of COVID-19-related encephalopathy/encephalitis (*n* = 27 CSF investigations),^41^ no antibodies against neuronal antigens were found. Specific details of the indirect immunofluorescence assays were not reported.

Our commercial assays detected weakly positive NMDAR, CASPR2 and LGI1 antibodies in the serum of four patients, but none were confirmed on tissue-based assays, and no patient fulfilled consensus criteria for antibody-mediated encephalitis.^40^ It is important to distinguish between anti-neuronal binding in tissue- and neuronal-based assays of any sort on one hand and clinically relevant anti-neuronal binding on the other hand, as our findings could be due to universal autoimmune reactions in patients with a severe systemic viral infection and universally increased IgG antibody levels (similar to, e.g. finding GAD65 or TPO antibodies as an epiphenomenon in patients with autoimmune encephalitis).^43^

In line with previous COVID-19 CSF studies,^14-18,23,24,41,44,45^ we found no signs of SARS-CoV-2 RNA in the CSF and no signs of intrathecal antibody synthesis. CSF SARS-CoV-2 antibodies against the spike protein were frequent, which could be due to a permeable BBB or changes in the CSF flow^34^ rather than a genuine neurotropic infection. The BBB becomes more permeable during critical illness,^46^ peripheral inflammation^47^ and is associated with an elevated Q-Alb,^33,34^ which are all variables that in our study correlated with increasing CSF SARS-CoV-2 antibody concentrations. Therefore, a disrupted BBB could result in the diffusion of systemic inflammatory molecules across the BBB that enter the CNS and contribute to neurological and neuropsychiatric symptoms observed in COVID-19.^48^ A recent study showed increased SARS-CoV-2 nucleoside antigen concentrations in the CSF of COVID-19 patients, which was associated with CNS immune activation,^44^ suggesting that the SARS-CoV-2 nucleocapsid or spike protein could trigger a CNS immune response. Indeed, SARS-CoV-2-derived and isolated spike protein S1 may cross the BBB^49^ and cause cognitive deficits, anxiety-like behaviour and hippocampal neuronal death in mice.^50^ However, we only measured antibodies against the spike protein, and it is unknown if these antibodies reflect the presence of the spike protein S1 in the CSF. However, not all CSF samples that met the manufacturer’s cut-off value for a positive SARS-CoV-2 antibody titre were indicative of a dysfunctional BBB as measured by Reiber’s formula^33,34^ (e.g. the CSF of one long-COVID patient had markedly elevated SARS-CoV-2 antibodies in CSF 10 months after COVID-19 symptom onset). This observation of SARS-CoV-2 antibodies in the CSF in individuals with an intact BBB could also be explained by passive diffusion of IgG into the CSF,^34^ which would correlate with increasing levels of SARS-CoV-2 IgG in serum. Finally, this highlights the importance of calculating the specific intrathecal synthesis index to discriminate between true viral neurotropism and blood-derived IgG.

### Strengths and limitations

Strengths include prospective data and sample collection, the inclusion of a broad range of mildly, severely and critically affected COVID-19 patients with neurological and neuropsychiatric symptoms, and thorough laboratory investigations with both commercial cell-based assays of known anti-neuronal antibodies and indirect immunofluorescence of brain-tissue-based assays. However, the broad range of COVID-19 severity and different neurological mechanisms that characterize our study population, also limits the study’s generalizability. Further limitations include the relatively small sample size, the lack of a prospective control group and the difficulty of conducting studies that require invasive procedures in acute patients during the COVID-19 pandemic, which resulted in varying sample sizes of blood and CSF.

## Conclusions

In this prospective study of mildly, severely and critically ill COVID-19 patients with neurological and neuropsychiatric manifestations, CSF SARS-CoV-2 antibodies were frequent, probably due to an impaired blood–brain barrier because we found no evidence of viral RNA or intrathecal antibody synthesis. Few patients had anti-neuronal CSF antibodies measured by commercial assays with no clinical correlate to a known syndrome of antibody-mediated autoimmune encephalitis. Overall, non-specific effects of critical illness (e.g. BBB dysfunction), rather than specific autoimmune neuronal injury, are more likely to be responsible for neurological and neuropsychiatric symptoms following COVID-19.

## Supplementary Material

fcad274_Supplementary_Data

## Data Availability

Anonymized data are available upon reasonable request from qualified investigators.
