# Correction to: SARS-CoV-2 and autoantibodies in the cerebrospinal fluid of COVID-19 patients: prospective multicentre cohort study

**DOI:** 10.1093/braincomms/fcae214

**Published:** 2024-07-02

This is a correction to: Vardan Nersesjan, Moshgan Amiri, Anna Christine Nilsson, Christian Wamberg, Veronika Vorobieva Solholm Jensen, Charlotte Bjerg Petersen, Anne-Mette Hejl, Anne-Mette Lebech, Anna Marie Theut, Charlotte Sværke Jørgensen, Morten Blaabjerg, Michael E Benros, Daniel Kondziella, SARS-CoV-2 and autoantibodies in the cerebrospinal fluid of COVID-19 patients: prospective multicentre cohort study, *Brain Communications*, Volume 5, Issue 5, 2023, fcad274, https://doi.org/10.1093/braincomms/fcad274

The following corrections have been made to the originally published manuscript:

1. RU/mL has been corrected to RU/L in:
  - Table 2. “*Serum (RU/mL), median (IQR)*” is now “*Serum **(RU/L)**, median (IQR)*”
  - Figure 1, A and B section. Serum labeled (RU/ml) is now **(RU/L)**
  - Graphical abstract, lower left panel, X-axis, Serum labeled (RU/ml) is now **(RU/L)**
2. Page 4 in pdf version: Equation written as “*0.93 [Q-Alb^2^ + 6 × 10^6^]^0.5–1.7^) × 10^−3^*” has been corrected to: “*0.93 [Q-Alb^2^ + 6 × 10^6^]^0.5^**–1.7)** × 10^−3^*”
3. Page 4 in pdf version, the statistical method section. The sentence “… *WHO established criteria.*”. Reference 24 has been corrected to 25.
4. Table 2, reference number 32 after “*BBB*” has been moved to after “*elevated protein*”
5. Figure 1C. Unit on the x-axis has been corrected from “(cells*10^-6/L)” to “(cells*10^9/L)”

These errors have been corrected in the original paper.

