# Supplementary material for: SARS-CoV-2 and autoantibodies in the cerebrospinal fluid of COVID-19 patients: prospective multicentre cohort study
Source: Brain Commun. 2023 Oct 17;5(5):fcad274. doi: 10.1093/braincomms/fcad274 (PMC10613856; doi:10.1093/braincomms/fcad274)
Supplement: fcad274_Supplementary_Data [file fcad274_supplementary_data.pdf]

Supplementary material:

SARS-CoV-2 and auto-antibodies in the cerebrospinal fluid of COVID-19 patients: prospective multicenter cohort study

Authors:

Vardan Nersesjan[1,2,3]; Moshgan Amiri[2]; Anna Christine Nilsson[4]; Christian Wamberg[5]; Veronika Vorobieva Solholm Jensen[6]; Charlotte Bjerg Petersen[7]; Anne-Mette Hejl[7]; Anne-Mette Lebech[8,9]; Anna Marie Theut[7]; Charlotte Sværke Jørgensen[6]; Morten Blaabjerg[10]; Michael E. Benros[1,2,*]; and Daniel Kondziella[2,9,*]

Affiliations:

1) Biological and Precision Psychiatry, Copenhagen Research Center for Mental Health, Mental Health Centre Copenhagen, Copenhagen University Hospital, Copenhagen, Denmark

2) Department of Neurology, Rigshospitalet, Copenhagen University Hospital, Copenhagen, Denmark

3) Faculty of Health and Medical Sciences, Department of Immunology and Microbiology, University of Copenhagen, Copenhagen, Denmark

4) Department of Clinical Immunology, Odense University Hospital, Odense, Denmark

5) Department of Anesthesia and Intensive Care, Bispebjerg and Frederiksberg Hospital, Copenhagen University Hospital, Copenhagen, Denmark

6) Virus and Microbiological Special Diagnostics, Statens Serum Institut, Artillerivej 5, Copenhagen S, Denmark

7) Department of Neurology, Bispebjerg and Frederiksberg Hospital, Copenhagen University Hospital, Copenhagen, Denmark

8) Department of Infectious Diseases, Rigshospitalet, Copenhagen University Hospital, Copenhagen, Denmark

9) Department of Clinical Medicine, University of Copenhagen, Copenhagen, Denmark.

10) Department of Neurobiology Research, Institute of Molecular Medicine, University of Southern Denmark, 5000 Odense, Denmark

*** These authors share last and corresponding authorships**

Supplementary table 1. Overview of available data of study outcomes

Outcome	No. of cases with available data / total study population
**Routine analysis**	
CSF leukocytes, x 10^6/L	38 / 38
CSF protein, g/L	38 / 38
CSF albumin, mg/L	38 / 38
CSF glucose,	38 / 38
CSF IgG, mg/L	21 / 38
**Microbiological analysis**	
**SARS-CoV-2 RNA via PCR**	
CSF	30 / 38
Serum	30 / 38
**SARS-CoV-2 IgG Ab against spike protein WANTAI**	
CSF	31 / 38
Serum	31 / 38
**SARS-CoV-2 IgG Ab against spike protein EUROIMMUN**	
CSF	34 / 38
Serum	32 / 38
HSV, VZV and EV DNA/RNA PCR	
CSF	30 / 38
Serum	30 / 38
**Autoimmune analysis**	
**CBA commercial**	
CSF	38 / 38
Serum	34 / 38
**TBA commercial**	
CSF	38 / 38
Serum	34 / 38

Abbreviations: CSF, cerebrospinal fluid; PCR, polymerase chain reaction; Ab, antibody; HSV, herpes simplex virus; VZV, varicella-zoster virus; EV, enterovirus; CBA, cell-based assay; TBA, tissue-based assay.

Supplementary table 2. Clinical and laboratory characteristics of COVID-19 patients with detected anti-neuronal antibodies in serum with commercial cell-based assays

Case	Clinical Phenotype	CSF findings		Commercial CBA		Commercial TBA		EEG	Brain CT / MRI
		Leuko. (E6/L)	Protein (g/L)	Serum	CSF	Serum	CSF		
#1	Peripheral facial palsy 17 days after pos. PCR	3	0.30	**CASPR2^{+}**	Neg.	Neg.	Neg.	NA	NA
#2	Prolonged fatigue and cognitive deficits	2	0.22	**LGI1^{+}**	Neg.	Neg.	Neg.	NA	Normal MRI
#3	Encephalopathy, followed by coma and death	2	0.50	**CASPR2^{+}**	Neg.	Neg.	Neg.	Encephalopathic slowing	Pontine and mesencephalic hypodensity on CT
#4	Encephalopathy, followed by coma and death	4	0.27	**NMDAR^{+}**	Neg.	Neg.	Neg.	Encephalopathic slowing	Normal CT

All positive serum findings were weakly positive.

Abbreviations: CBA, cell-based assay; CSF, cerebrospinal fluid; CT, computerized tomography; CASPR2, contactin-associated protein-like 2; LGI1, leucine-rich glioma inactivated 1; Leuko, leukocytes; MRI, magnetic resonance imaging; EEG, electroencephalogram; NMDAR, N-methyl-D-aspartate receptor; NA, not applicable; PCR, polymerase-chain-reaction for SARS-CoV-2; TBA, tissue-based assay.
